# Stable learning of functional maps in self-organizing spiking neural networks with continuous synaptic plasticity

**DOI:** 10.3389/fncom.2013.00010

**Published:** 2013-02-27

**Authors:** Narayan Srinivasa, Qin Jiang

**Affiliations:** Center for Neural and Emergent Systems, HRL Laboratories LLCMalibu, CA, USA

**Keywords:** spiking networks, STDP, learning, functional maps, orientation selectivity, ocular dominance, stability

## Abstract

This study describes a spiking model that self-organizes for stable formation and maintenance of orientation and ocular dominance maps in the visual cortex (V1). This self-organization process simulates three development phases: an early experience-independent phase, a late experience-independent phase and a subsequent refinement phase during which experience acts to shape the map properties. The ocular dominance maps that emerge accommodate the two sets of monocular inputs that arise from the lateral geniculate nucleus (LGN) to layer 4 of V1. The orientation selectivity maps that emerge feature well-developed iso-orientation domains and fractures. During the last two phases of development the orientation preferences at some locations appear to rotate continuously through ±180° along circular paths and referred to as pinwheel-like patterns but without any corresponding point discontinuities in the orientation gradient maps. The formation of these functional maps is driven by balanced excitatory and inhibitory currents that are established via synaptic plasticity based on spike timing for both excitatory and inhibitory synapses. The stability and maintenance of the formed maps with continuous synaptic plasticity is enabled by homeostasis caused by inhibitory plasticity. However, a prolonged exposure to repeated stimuli does alter the formed maps over time due to plasticity. The results from this study suggest that continuous synaptic plasticity in both excitatory neurons and interneurons could play a critical role in the formation, stability, and maintenance of functional maps in the cortex.

## Introduction

The spatial and temporal properties of a distributed pattern of neural activity in V1 with differentially tuned responses of individual neurons to features of visual space such as orientation, spatial frequency, and direction of motion were first recognized by Hubel and Wiesel (1962, 1963, 1968, 2005). Using microelectrodes and neuroanatomical tracers, they established that the neural activity of a population of neurons with such differentiation represented *functional maps*. This seminal work has inspired a large body of subsequent research to understand the properties of these functional maps and its relation to cortical function as well as to understand the mechanisms by which these maps are formed during development (Blakemore and Cooper, 1970; Miller, 1996; Rao et al., 1997; Buonamano and Merzenich, 1998; Miller et al., 1999; Yuste and Sur, 1999; Basole et al., 2003, 2006; Hensch, 2005; Schummers et al., 2005; Yu et al., 2005; Shapley et al., 2007; White and Fitzpatrick, 2007; Huberman et al., 2008; Xing et al., 2011).

Two types of functional maps have been particularly well explored. The first map called the ocular dominance maps, or ODM, is based on interactions between axons of the neurons in the lateral geniculate nucleus (LGN) and neurons in layer 4 of V1. Here clusters of thalamocortical axon terminals that serve the left or right eye are organized in layer 4 via the topological relations established in the LGN to form ODMs. The second functional map called the orientation selectivity map, or OSM, is a map of orientation preference that is elaborated with a high degree of selectivity in V1 but not in the LGN. A key mechanism implicated in the formation of these functional maps during development is activity-dependent plasticity (Purves and Lichtman, 1985; Katz and Shatz, 1996; Ruthazer and Stryker, 1996; Crair et al., 1998; Crowley and Katz, 1999).

A number of *in vitro* experimental studies (Levy and Steward, 1983; Magee and Johnston, 1997; Markram et al., 1997; Bi and Poo, 1998; Debbane et al., 1998; Caporale and Dan, 2008) suggest that repeated pairing of pre- and postsynaptic activity in the form of action potentials, or spikes, can lead to long-term changes in synaptic efficacy. The sign and magnitude of the change in synaptic efficacy depends upon on the relative timing between the pre- and postsynaptic spikes and is known as spike-timing-dependent plasticity (STDP). STDP is now a well-established physiological mechanism of activity-driven synaptic regulation *in vivo* as well as observed in the Xenopus visual system (Mu and Poo, 2006; Vislay-Meltzer et al., 2006), the locust mushroom body (Cassenaer and Laurent, 2007), and rat visual (Meliza and Dan, 2006) and barrel (Jacob et al., 2007) cortex. STDP has also shown to have better explanatory power than more conventional Hebbian correlation-based plasticity at explaining both cortical reorganization in cat primary visual cortex (Young et al., 2007) and connectivity in locust olfactory system (Finelli et al., 2008). The STDP is a local learning rule that forces synapses to compete such that the spiking activity of a post-synaptic neuron becomes selective to a small subset of pre-synaptic input spikes. This feature was exploited in some spiking models to demonstrate map development (Bartsch and van Hemmen, 2000; Song and Abbott, 2001; Billings and van Rossum, 2009).

In the present study a spiking model is described that provides a plausible set of mechanisms based on STDP for the formation and maintenance of ODMs and OSMs. This developmental model simulates functional map formation during three phases: an early experience-independent phase, a late experience-independent phase, and a subsequent refinement phase during which experience acts to shape map properties. There are other models that have also modeled developmental phases of functional maps in the visual cortex (Sirosh and Miikkulainen, 1995; Bauer et al., 2000; Swindale, 2000; Bednar and Miikkulainen, 2004; Yang et al., 2012). However, we present a spiking model that employs STDP as the basis to form and stabilize functional maps across the three phases of development. The resulting ODMs represent V1 neurons that show eye selectivity in response to two sets of monocular inputs from the LGN. The OSMs on the other hand feature pin wheel-like patterns to represent orientation preference in a smooth and continuous fashion. However, point discontinuities that appear at the center of these pin wheels in animal data (Maldonado et al., 1997) is not found in our model simulations. It also contains other forms of discontinuities such as fractures and breaks as will be defined in the next section.

## Materials and methods

For the purpose of simplicity and clarity, the focus of this study will be on the dynamics of interaction between the neurons in LGN and layer 4 in V1. The model is developed in three phases where the first and second phases correspond to a *pre-critical* period while the third phase corresponds to a *critical* period (Hensch, 2005). In the early experience-independent phase, spontaneously generated neuronal activity in the cortex and LGN facilitates activity-dependent plasticity and formation of OSMs and ODMs (Wiesel and Hubel, 1974; Chapman et al., 1996; Crair et al., 1997; Ferster and Miller, 2000; Trachtenberg et al., 2000; Chiu and Weliky, 2001; Huberman et al., 2006). This is followed by a late-experience-independent phase where interactions between LGN and layer 4 are driven by the influence of retinal waves (Godfrey and Swindale, 2007) to enable the refinement of ODMs and OSMs (Crair et al., 1997; Crowley and Katz, 1999, 2002; Butts, 2002; Katz and Crowley, 2002; Huberman et al., 2006; Feller, 2009). In the final experience-dependent phase, neuronal activity is driven by natural visual stimuli from the environment that drives the maturation of the already formed ODMs and OSMs (Crair et al., 1998; Sengpiel et al., 1999; White et al., 2001; Coppola and White, 2004; Smith and Trachtenberg, 2007; White and Fitzpatrick, 2007). It should be noted that the model results are meant to show that the maturation of the formed OSMs and ODMs are driven by activity-dependent plasticity while qualitatively simulating some of the process constraints (such as the influence of retinal waves) during various stages of development. These maps have some similar qualitative properties as those found in animals but this does not imply actual adherence to the maps or process in any particular species.

### Model architecture

The spiking model architecture in this study assumes that the initial structure of connections despite being random and local in nature is nevertheless present from the beginning. It is known that this initial formation of the map depends upon molecular gradients that serve as guides for axons to topologically appropriate portions of the map (Yuste and Sur, 1999; Crowley and Katz, 2002; Hensch, 2005; Taha and Stryker, 2005; Huberman et al., 2006; White and Fitzpatrick, 2007). The model also assumes that the neurons are mature unlike in reality where neurons are immature during very early stages of development and are characterized by a high concentration of Cl^−^ ions as a result of which all neurons are depolarizing (Hensch, 2005).

**Figure 1:**
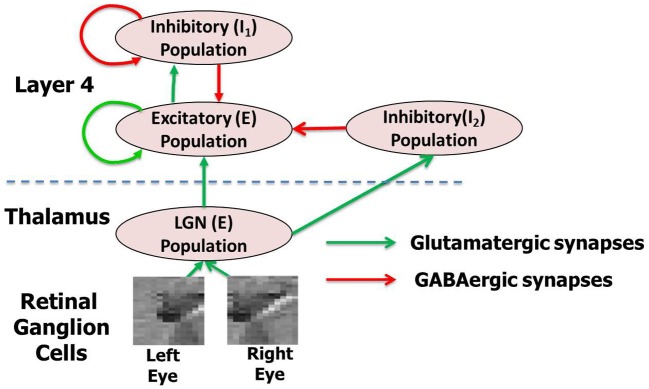
**The complete network model (A) with thalamocortical circuit where thalamic afferents from the LGN activate the principal cells of layer 4 of V1 via geniculocortical synapses.** The layer 4 and LGN are both modeled as an *E-I* network. There are two inhibitory populations in layer 4: the feedback inhibitory population *I*_1_, which does not receive any inputs from LGN but only from the *E* neurons of layer 4 and the feedforward inhibitory population *I*_2_, which does. The LGN receives spikes from retinal ganglion cells (RGC). The LGN and layer 4 neurons in the model are separated by the dashed line in the figure. This complete model is used for simulating the experience-dependent phase of development. For each network layer, 60% of randomly chosen neurons are injected with background noise in form of currents (*I*_inj_) for 30 ms. A new of set of 60% randomly chosen neurons at all layers are selected again after that and are injected with background noise. This process is repeated throughout all three phases of development. For simulating early experience-independent phase (Phase 1) the complete network is purely driven by the background noise at both LGN and layer 4. For simulating late experience-independent phase (Phase 2) the LGN is activated by spikes due to retinal waves from RGC.

The model is designed to address the thalamocortical circuit where thalamic afferents from the LGN activate the principal cells of layer 4 of V1 via geniculocortical synapses (Antonini and Stryker, 1978; Ursey et al., 1999; Yuste and Sur, 1999; Bartsch and van Hemmen, 2000). For convenience, the principal cells within layer 4 will be referred to as *E* neurons while all the inhibitory interneurons will be referred to as *I* neurons throughout the article. The *E* neurons are connected to other local *E* neurons and *I* neurons to form a dense local recurrent network. In our model, the *E* and *I* neurons will make up two sub-layers within layer 4. Similarly the LGN in the full model architecture (Figure 1) is also modeled as an Excitatory-Inhibitory (*E-I*) network. The *E-I* network model is a commonly used design to simulate models of thalamocortical areas (Binzegger et al., 2004; Kremkow et al., 2007; Kumar et al., 2008). The synapses in the model are plastic throughout all phases of development (Hensch, 2005) and the self-organization process refers to the change in the synaptic conductance during development.

**Figure 2:**
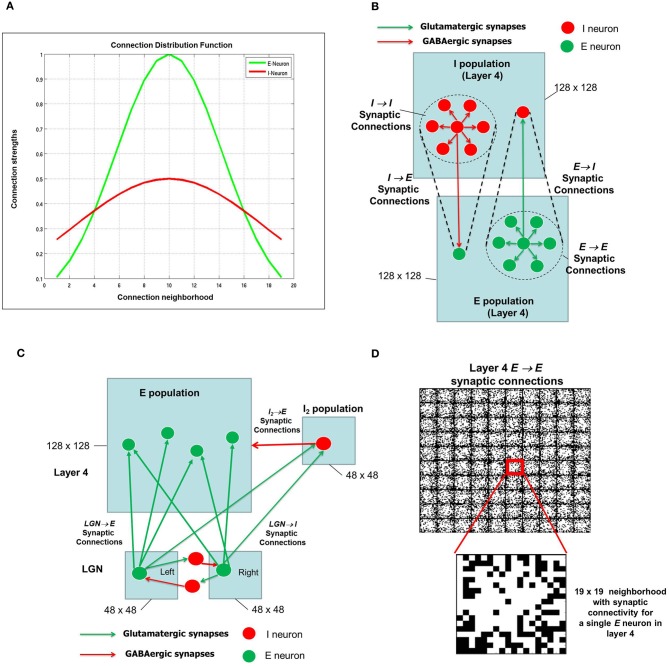
**The synaptic connectivity density distribution for the network. (A)** From any *E*-neuron to any other neuron (*E* or *I*) in shown in green and from any *I*-neuron to any other neuron (*E* or *I*) is shown in red. **(B)** There are four types of synapses depending on the pre- and post-synaptic neuron: *E* → *E*, *E* → *I*, *I* → *E*, and *I* → *I*. It should be noted that the network has periodic boundary conditions such that topmost and bottommost neurons are regarded as neighbors, as are the leftmost and rightmost columns within each layer. The neighborhood around each *E* or *I* neuron is shown as a dotted circle. **(C)** The LGN network is also an *E-I* network as shown here with mutually inhibiting connections between neurons that receive inputs from the RGCs (not shown) from the left and right eye. Each LGN neuron from both eyes project to a neuron and its neighborhood in layer 4. For convenience, only one such projection is shown here. The LGN network (2 × 48 × 48) is smaller than the layer 4 network (128 × 128). In addition, the LGN inputs from the left and right eye populations project to the *I*_2_ population in layer 4 which consists of 48 × 48 inhibitory neurons as well. **(D)** The synaptic connections for a set of 10 × 10 *E* neurons in layer 4 are shown here. The red square shows a single *E* neuron with a 19 × 19 neighborhood. The white pixels within each such square indicate synaptic connections with maximum synaptic strength while black pixels indicate synaptic connections with zero synaptic strength. A closer look at the 19 × 19 neighborhood for one of *E*-neuron shows the initial strengths of synapses from the *E*-neuron in the center to its neighboring *E* neurons. These synaptic strengths are randomly distributed.

In general, neurons in the cerebral cortex are densely connected to neurons close to it and sparsely connected to neurons far away from it (Schummers et al., 2004; Song et al., 2005; Perin et al., 2011; Voges and Perrinet, 2012). In particular, models of cortical function often assume that cortical circuitry acts in a center–surround fashion, whereas separated pairs of cells have a mutually suppressive influence. Further, to make the enhanced cortical patterns congruent with the sensory representation of the system, the cortical interactions must depend on the functional distance between the cells, determined by the features coded by them. This functional circuitry, known as “Mexican hat” organization, has been adopted in network models of orientation selectivity (Somers et al., 1995; Kang et al., 2003). In our model, we make a similar assumption where the Gaussian distribution of synaptic connections for the excitatory or glutamatergic synapses is dense and narrow in its spatial extent (Figure 2A) compared to the inhibitory or GABAergic synapses which are more broadly distributed.

The *E* neurons in layer 4 (Figure 2B) are connected to its neighboring *E* neurons and *I* neurons in *I* layer of layer 4. Similarly, *I* neurons in layer 4 are connected to its neighboring *I* neurons as well as the *E* neurons in layer 4. Thus there are four types of synapses depending on the pre- and post-synaptic neuron: *E* → *E*, *E* → *I*, *I* → *E*, and *I* → *I*. The first two types of synapses are excitatory in nature and obey E-STDP rule while the last two synapses are inhibitory in nature and obey the I-STDP rule for plasticity (Woodin et al., 2003; Caporale and Dan, 2008; Hartmann et al., 2008). The synaptic connections are initialized with random synaptic strengths and obey the connection density as prescribed by the Gaussian distribution shown in Figure 2A for all four types of synapses. An example of a 10 × 10 set of *E* neurons in layer 4 with *E* → *E* synaptic connectivity is provided in Figure 2D. Without loss of generality, the *E* and *I* layers in layer 4 in all our simulations will consist of a 2-D sheet of 128 × 128 neurons. The LGN in the full model (Figure 1) is also composed of an *E-I* network similar to layer 4. The *E* and *I* layers within the LGN consist of 48 × 48 neurons and there are two such *E*-*I* networks in the LGN corresponding to each eye. Each LGN *E* neuron from both the eyes makes sparse and random synaptic connections to *E* neurons in layer 4 (Figure 2C). In addition to these LGN → *E* synaptic connections, the LGN neurons are also connected to a feedforward inhibitory population of neurons which provide feedforward inhibition to the same *E* neighborhood in layer 4. Feedforward inhibition is known to play a role in input normalization and expansion of cortical dynamic range (Pouille et al., 2009). The ultimate distribution of synaptic strengths in model synapses is dictated by E-STDP and I-STDP during the developmental process.

The spiking model simulations were performed using the HRLSim (Minkovich et al., under revision) which is a multiple graphical processing unit (GPU) based spiking simulator in C^++^. This simulator is an extension of a single GPU developed previously (Nageswaran et al., 2009) and uses an MPI interface and other optimizations to enable scalable and real-time simulation of large scale spiking neural networks. The computations to estimate the ODM and OSM were performed in MATLAB. The details of the neuronal and synaptic mechanisms and the performance metrics and measures used for the various experiments are now provided.

### Neuron model

In this study the leaky integrate and fire neuron model (Vogels et al., 2005) is used where each neuron receives multiple input current signals and the dynamics of its membrane potential *V* can be determined as:

(1) $$\tau_{m}\frac{dV}{dt}=(V_{\text{rest}}-V)+\sum \text{​}w(t)(E_{\text{ex}}-V)-\sum \text{​}z(t)(E_{m}-V)+I_{\text{inj}}$$

when *V* reaches a threshold voltage *V*_*T*_, the neuron fires a spike, and *V* is reset to *V*_reset_. This basic model provides several control variables for the membrane voltage including synaptic conductance *w* (excitatory) and *z* (inhibitory), membrane time constant τ_*m*_, reversal potentials *E*_ex_ and *E*_in_, and resting voltage *V*_rest_. The parameter *I*_inj_ represents the current injected into the neuron. Synaptic inputs to the neuron are modeled as conductance changes where a single pre-synaptic spike at time *t* generates a synaptic conductance for excitatory and inhibitory synapses as follows:

(2) $$w(t)=we^{\frac{-t}{\tau_{\text{AMPA}}}}$$

(3) $$z(t)=ze^{\frac{-t}{\tau_{\text{GABA}}}}$$

where the time constants τ_AMPA_ and τ_GABA_ are used to model the kinetics of AMPA and GABA receptors. The value of the excitatory and inhibitory synaptic conductance *w* and *z* in Equation (1) is determined by STDP. Table 1 provides a list of values for all the constants used to simulate the neuron model. During model simulations, the continuous membrane equation is updated using Euler integration with a time step of 1 ms.

**Table 1:** **Neuron parameters**.

**Parameter**	**Value**
τ_*m*_	20 ms
E_Ex_	0.0 mV
E_inh_	0.0 mV
V_reset_	−60 mV
V_rest_	−74 mV
V_*T*_	−54 mV
τ_AMPA_	10 ms
τ_GABA_	50 ms

### Excitatory STDP

**Figure 3:**
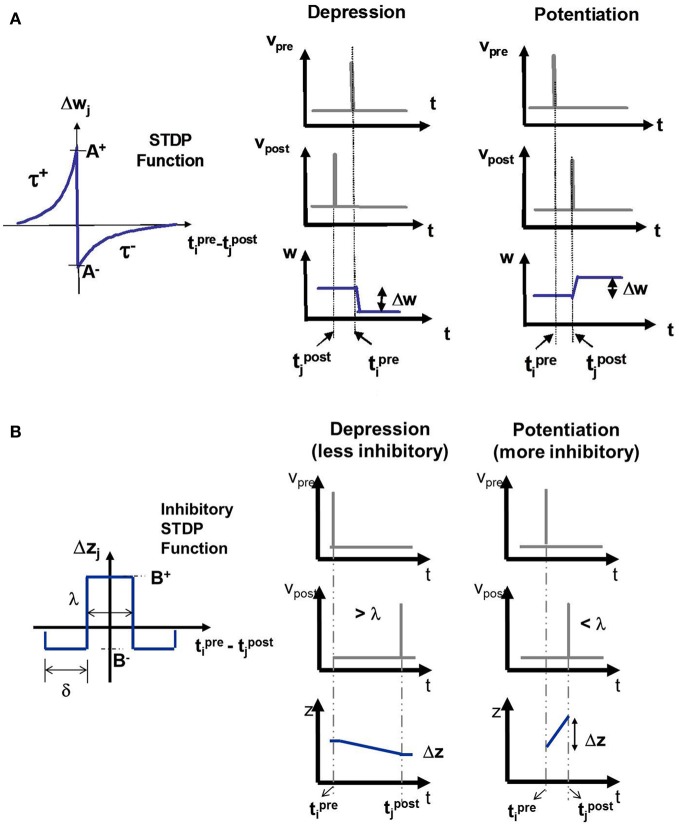
**(A)** The E-STDP function modulates the excitatory synaptic conductance *w* based on the timing difference (*t*^pre^_*i*_ − *t*^post^_*j*_) between the action potentials of pre-synaptic neuron *i* and post-synaptic neuron *j*. The control parameters (*A*^+^, *A*^−^, τ^+^, and τ^−^) can be used to modify the amount of potentiation and depression (see “Materials and Methods”). **(B)** The I-STDP function modulates the inhibitory synaptic conductance *z* based on the timing difference (*t*^pre^_*i*_ − *t*^post^_*j*_) between the action potentials of pre-synaptic neuron *i* and post-synaptic neuron *j* (see “Materials and Methods”). If the timing difference is > λ, then the synapses become less inhibitory and the change itself is of a smaller magnitude while it is the opposite case when the timing difference is <λ.

The E-STDP function modulates the excitatory synaptic conductance *w* based on the timing difference (*t*_pre_ − *t*_post_), or Δ*t*, between the spike times of pre- and post-synaptic neuron (Figure 3A). The control parameters (*A*^+^, *A*^−^, τ^+^, τ^−^) can be used to modify the amount of potentiation and depression (Table 2). The synaptic conductance is computed as:

(4) $$w_{\text{new}}=w_{\text{old}}+\Delta w$$

where

(5) $$\Delta w=g_{\max}\times F(\Delta t)$$

and

(6) $$F(\Delta t)=\left\{ \begin{array}{cc} A_{+}\times \exp\left(\frac{\Delta t}{\tau_{+}}\right), & \text{if }\Delta t<0\\ -A_{-}\times \exp\left(\frac{\Delta t}{\tau_{-}}\right), & \text{if }\Delta t\geq 0 \end{array}\right.$$

If *w*_new_ > *g*^*E*^_max_, then *w*_max_ = *g*^*E*^_max_. On the other hand if *w*_new_ < 0, then *w*_new_ = 0. The factor β = |*A*^−^ τ^−^|/|*A*^+^ τ^+^| controls the relative amounts of depression to potentiation during learning.

**Table 2:** **STDP parameters**.

**STDP**	**Parameter**	**Value**
**E-STDP**		
	g^*E*^_max_	0.035 nS
	A^+^	0.0 mV
	β	1.02 nS
	τ^+^	20 ms
	τ^−^	20 ms
**I-STDP**		
	g^*l*^_max_	0.0035 nS
	B^+^	0.045 nS
	B^−^	0.025 nS
	λ	12 ms
	δ	40 ms

### Inhibitory STDP

The I-STDP function modulates the inhibitory synaptic conductance *z* based on the timing difference Δ*t* between the spike times of pre- and post-synaptic neuron (Figure 3B). The synaptic conductance is computed as:

(7) $$z_{\text{new}}=z_{\text{old}}+\Delta z$$

The change Δ*z* = *B*^+^ is more inhibitory when −λ ≤ Δ*t* ≤ λ. The change Δ*z* = *B*^−^ is less inhibitory when −λ −δ ≤ Δ*t* < −λ or λ < Δ*t* ≤ λ + δ. There is no change in inhibition if either Δ*t* ≤ −λ −δ or Δ*t* ≥ λ + δ. If *z*_new_ < 0 then *z*_new_ = 0. On the other hand, if *z*_new_ > *g*^*I*^_max_ then *z*_new_ = *g*^*I*^_max_. The control parameters (B^+^, B^−^, λ, and δ) (see Table 2) can be used to vary the relative amounts of potentiation and depression during learning.

### Model synaptic connectivity

The synaptic connectivity in layer 4 and LGN is initialized using a Gaussian density function as:

(8) $$p(x,y)=\alpha \times e^{\frac{-[{\left(x-x_{0}\right)}^{2}\;+{\left(y-y_{0}\right)}^{2}]}{2\times \sigma }}$$

The point (*x*_0_, *y*_0_) represents the center neuron and (*x*, *y*) represents the position of neurons within its neighborhood. The constants α and σ (Table 3) control the maximal distribution probability and distribution variation.

**Table 3:** **Other model parameters**.

**STDP**	**Parameter**	**Value**
**SYNAPTIC CONNECTIVITY**		
	σ_*E*_	1.0
	σ_*l*_	0.5
	α_*E*_	2.5
	α_*l*_	3.5
	τ^−^	20 ms
**BACKGROUND AND NOISE**		
	A	1.07
	Δ*t*	30 ms
	μ	0.65
	t_1_	0 s
	t_2_	6000 s
**OSM COMPUTATION**		
	ϕ	0°, 45°, 90°, 135°
	σ_*a*_	2.5
	σ_*b*_	5.7
	ϒ	[−8.0, 8.0]

### Background signal and noise

A random voltage injection is used to mimic cortical input spikes generated from brain regions (or background) as well as noise. The injected voltage *V*_inj_(*t*) is modeled as:

(9) $$V_{\text{inj}}(t)=Au(r),\;t\in [t_{1},t_{1}+\Delta t]$$

with

(10) $$u(r)=\left\{ \begin{array}{cc} 1 & r\geq \mu \\ 0 & \text{otherwise} \end{array}\right.$$

The constant *A* determines the amplitude of voltage injection. The variable *r* is a random variable uniformly distributed in the range (0, 1.0). The parameter Δ*t* (Table 3) is the voltage injection duration while the parameter μ represents the percentage of neurons that receive voltage injection randomly. Thus, the injection time duration Δ*t* is used to set an injection frequency. In our model, before the eyes open, the spiking activity of neurons is driven primarily by random current injection *I*_inj_ (in Equation 1) by multiplying *V*_inj_*(t)* into currents using a fixed synaptic conductance constant of 0.00125 nS.

### Retinal wave model

Before the onset of stimulus driven activity, which helps refine neural organization in later developmental stages, neural circuits generate spontaneous patterns of activity which guide early development (Katz and Shatz, 1996). In the retina, spontaneous activity takes the form of coordinated bursts of spikes in the neighboring retinal ganglion cells (RGC) that slowly spread across the retina. They can initiate at any retinal location and cover the entire retina in minutes (Schiller, 1992; Butts, 2002; Godfrey and Swindale, 2007; Feller, 2009). The retinal waves were generated in our model as follows. The retina is assumed to have 48 × 48 neurons or RGCs. For each eye, a randomly chosen set of *N* RGCs are selected for retinal wave initiation. For each initiation site, a direction *d* is selected for wave propagation from a set of eight possible directions (i. e., 0°, 45°, 90°, 135°, 180°, 225°, 270°, and 315°). At each initiation site, RGCs within a neighborhood of size 10 × 10 are then activated at the first time step (*t* = 1 ms). The strength of the stimulation is varied in a Gaussian fashion where neighbors at closer distance are stimulated more strongly than neighbors that are further apart. In the next time step (*t* = 2 ms) the next RGC to be stimulated is selected after moving by two steps (i.e., velocity is 2 steps/ms) in the direction *d*. The newly stimulated RGC is used to activate RGCs within a 10 × 10 neighborhood and the process is continued. The total duration for each retinal wave for each eye is set to 10 ms. The activity of the RGCs is converted into spike trains as described below. The process is continued to generate several retinal waves during the late activity-independent developmental phase.

### Spike encoding of retinal waves and natural images

The input visual images are in the form of either retinal waves or other natural images. For natural images, we use the Caltech 101 image dataset (Fei et al., 2006). A total of two thousand 48 × 48 images were cropped from the database as follows. A subset of images was selected from the database such that the central portion of the image had some texture and contrast in them. These original images of size 320 × 200 were down-sampled to 128 × 128 images and then a 48 × 48 portion from the center of these images (see Figure 11) was extracted. These 48 × 48 images were provided as RGC inputs to the LGN after spike encoding the images as described below. While the Caltech 101 dataset was developed for object recognition purposes, the statistics of the images extracted is still representative of natural images in general.

We created stereo pairs from every image in the dataset by shifting the right eye image by a maximum of 10 pixels to the left or right and 10 pixels to the top or bottom with respect to the left image. This simulates disparity in the two images. In addition to disparities, we also scale the right image by a small scaling factor between 1 and 1.05 to simulate small perspective changes. Such affine transforms are commonly used in computer vision (Zhang and Xu, 1998).

The images are converted into spike sequences by an encoding process, and the sequences serve as input to neurons in the LGN layer. The encoding process is generated based on Poisson statistics where the Poisson distribution is used to generate the interspike interval (ISI) for each pixel. The mean value of pixel intensities in the image serves as the mean value of the Poisson distribution. The Poisson distribution is given as:

(11) $$P(x)=\frac{\lambda^{x}e^{-\lambda }}{x!}$$

where λ is the mean value of pixel intensities and *x* is the ISI. To generate the spikes for a given pixel at a given time, we randomly select a probability value *P*(*x*) at each time step and then compute *x* using Equation (11). This sets the time step at which the next spike is generated as part of the encoding process.

### Recurrent cortical map computation

The recurrent cortical map (RCM) computation is based on the synaptic conductances between *E* → *E* neurons in layer 4. The purpose of RCM computation is to identify evolution of local synaptic connectivity within the *E* sub-layer of layer 4 during the various phases of development. The RCM is estimated by using a Gaussian bar function as an orientation template (Bartsch and van Hemmen, 2000) to search for the best orientation match within its neighborhood. The *E* neurons are color coded based on the best match score. The resulting image of the color coded *E* neurons constitutes a RCM.

Assuming that an *E* neuron is located at *p*, the Gaussian bar function is given by:

(12) $$G_{xy}(\phi ,p)=\exp\left\{\frac{-{\left[x\cos\phi +y\sin\phi -\gamma \right]}^{2}}{2\times \sigma_{a}^{2}}\right\}\times \exp\left\{\frac{-{\left[y\cos\phi -x\sin\phi \right]}^{2}}{2\times \sigma_{b}^{2}}\right\}-G_{0}(\phi ,p)$$

where the variables *x* and *y* varies within *E*-neuron's locally connected neighborhood and ∑_*xy*_*G*_*xy*_ (ϕ,γ) = 0. For each of the four orientations, ϕ ∈ {0°, 45°, 90°, 135°}, the parameter γ is varied to determine the maximal orientation match as:

(13) $$R\left(\phi \right)=\max_{p}\left[\sum_{xy}w\left(x,y\right)G_{xy}\left(\phi ,\gamma \right)\right]$$

With the maximal orientation match, a direction vector $\vec{d}(\phi )=(R(\phi ),\;2\phi )$ is constructed for each orientation. Then, the four direction vectors of the four orientations are combined into a final direction vector as:

(14) $$\vec{S}=(R_{s},\phi_{s})=\vec{d}(0^{{}^{\circ}})+\vec{d}(45^{{}^{\circ}})+\vec{d}(90^{{}^{\circ}})+\vec{d}(135^{{}^{\circ}})$$

The orientation of synaptic weights for the *E*-neuron is determined by

(15) $$\phi_{\text{or}}=\phi_{s}/2$$

The RCM is obtained by color coding each *E* neuron in layer 4 using ϕ_or_. The RCMs does not reflect orientation selectivity of OSMs since they are not measured based on inputs from the LGN and only show anisotropies in the local pattern of connectivity within layer 4.

### ODM computation

The ODM was computed using the following procedure. Each *E* neuron in layer 4 receives inputs from *E* neurons in the LGN. The initial synaptic conductance values for all input synapses from LGN are set to the same value. Thus, the *E* neurons initially start off by responding equally to inputs from both the eyes and are thus labeled as binocular. These geniculocortical synapses are tuned using STDP during the three phases based on random activity, retinal waves and with natural stimuli. The number of afferent synapses into an *E* neuron of layer 4 after each phase of development is tracked from each eye and the *E* neuron is assigned a membership to one of the two eyes based on the eye for which the sum of synaptic conductances from LGN →*E* is greater. It should be noted that this eye selectivity naturally emerges without the need for setting any arbitrary thresholds due to two factors. The first factor is due to mutual inhibition between the LGN neurons corresponding to the left and right eye (see Figure 2C). I-STDP in these inhibitory synapses forces the LGN neurons to compete and this competition affects the ability of *E* neurons in LGN to influence the *E* neurons in layer 4. The second factor is that E-STDP in the synapses from LGN → *E* pathway also creates a competition between synapses from the two eyes (Miller et al., 1989). These two competitive factors combine to break the symmetry in the geniculocortical synapses from the two eyes by increasing the synaptic conductances for inputs from the eye that attains a positive bias and vice versa.

Over time, if there is some spatial structure in the inputs (as it happens after Phase 1), the synaptic conductances in the LGN → *E* pathway become more separated in magnitude based on the eye due to aforementioned competition until the difference becomes stable. This causes the inputs from one eye in the LGN to eventually dominate the response of *E* neurons in layer 4. The reason for the appearance of contiguous patches of eye preference is due to the stable formation of the RCM. This map indicates local structure in the lateral excitation of neighboring *E* neurons (see Figure 2B) in layer 4 to similar stimuli. Thus, as a given *E* neuron develops a preference for one eye due to bottom–up competition as described above, the neighboring *E* neurons in layer 4 also tend to receive excitation from this *E* neuron and thus can influence their preference as well over time such that a well-developed and stable ODM emerges.

In order to measure the emergence of eye selectivity during various phases of development, we compute the sum of synaptic conductances for the geniculocortical synapses from the left and right eye separately for each *E* neuron. We then compute the average and standard deviation of the difference between the two sums for all the *E* neurons in layer 4. If the ODMs are truly mature, then this average should be substantial in terms of the total dynamic range of the synaptic conductances while the standard deviation must be very small. Furthermore, these values must also stabilize over time indicating that the ODMs are both mature and stable. It should be noted that ODM maps generated in all phases are smoothed using a median filter of size 3 × 3 to remove small speckle noise due to some neurons developing opposite eye preference on occasion in a neighborhood of *E* neurons that have the same eye preference.

### OSM computation

For clarity, we follow the definition of orientation preference and orientation selectivity provided in Blasdel (1992). Orientation preference of an *E* neuron in layer 4 is the orientation that yields the strongest response while orientation selectivity is the rate at which responses fall to zero with increasing displacement from the preferred orientation. The orientation selectivity of *E* neurons in layer 4 cannot be computed using RCMs since that computation does not involve input stimuli from LGN to the *E* neurons of layer 4. In order to derive OSMs, we stimulate the LGN at a given location *p* within it (on both eyes) using an oriented rectangular bar with a length of 15 pixels and a width of 5 pixels. At the end of each phase of development, the oriented bar in a given orientation α is moved bilaterally by different amounts (each amount being less than <7 pixels) during odd and even trials within the LGN layers and the resulting spikes from the stimuli (Equation 1) are used to stimulate the *E* neurons that receive these spikes in layer 4. The firing rate of spikes generated by each *E* neuron in layer 4 in response to the LGN inputs is calculated by counting the number of spikes in a 10 s (or 10,000 steps) time window. The resulting spike activity provides a sense for which *E* neurons respond more strongly to the input spikes from LGN for a given bar orientation. In other words, it provides an estimate of orientation selectivity of the *E* neurons in layer 4 to that particular oriented bar in the LGN. This process is repeated for four different orientations of the bar (i. e., α = 0°, 45°, 90°, and 135°).

Using the firing rates of *E* neurons we then apply the algorithm described in Blasdel (1992) to compute OSM as follows. First, we compute the differential between positive and negative images corresponding to complimentary stimuli—for example, α = 0° and α = 90 ° would correspond to a positive and negative image. The negative image is subtracted from the positive and this reveals the change in the response to the stimulation due to the horizontal and vertical bar in the LGN. The differential image is then transformed at every location, in every image, into vectors displayed either as cosine and sine pairs, in Cartesian coordinates, and as magnitudes and angles in polar coordinates with angles corresponding to twice the positive stimulus orientation, and lengths corresponding to net intensities. The resulting vectors from the transformed images are added to reflect orientation-weighted contributions from each image. Since stimulus orientations are multiplied by two, contributions generated by similar orientations reinforce one another, while those generated by orthogonal orientations cancel. The polar coordinates (angles and magnitudes) that are computed from the summed image reflect the orientation preference and selectivity of the *E* neurons in layer 4 (Blasdel, 1992; Miikkulainen et al., 2005).

The orientation preferences are analyzed with respect to their gradient that measures the rate of change at every point in two dimensions. Following Blasdel, we compute the gradient in *x* and *y* and then convert the result to Polar coordinates. The magnitude of the gradient calculated from the polar coordinates indicates the steepest rate of change at any point irrespective of direction. The gradient magnitude is used to find discontinuities which appear as short lines or dots running across a region of continuous tone (Blasdel, 1992). These discontinuities can signify either a *fracture* if it appears as a short line between two contiguous regions with a gradient of 90° or more, or signify *pinwheel formation*, if the gradient magnitude plot contains dots. This entire process described above was repeated after each developmental phase to assess the formation of OSM.

In order to get robust orientation gradients as well as orientation selectivity and preferences, the Blasdel approach averages the raw data from the summed Cartesian images (see Figure 13 in Blasdel, 1992). The purpose of this averaging process was to smooth out noise and improve the signal-to-noise ratio in these images. We smooth the summed *x* and *y* Cartesian images from our model using a median filter and then convert them into polar representation to obtain the orientation preference and selectivity of the *E* neurons. In order to estimate the correct filter size for smoothing, we adopt an iterative convergence process as follows. We first compute the orientation gradient responses for the raw OSMs. The raw OSM data in our model has small patches of noisy neuron orientation preference responses amidst regions of uniform orientation preference. The orientation gradients computed with such noise yields spurious discontinuities in the form of extraneous dots and lines. To mitigate this, we gradually increase the filter size of the median filter and repeat the above process to compute the orientation gradients until there are no spurious discontinuities due to those noisy neurons. This process does not change the resulting orientation preference maps in any qualitative fashion but filters out the noisy neuron preferences. We find that a median filter of 9 × 9 was sufficient to avoid any orientation gradient artifacts due to noisy orientation preferences. This approach was used to compute orientation selectivity, orientation preference and orientation gradients as well as perform analysis on the resulting functional maps after each phase of development.

While the smoothing operation during OSM computation based on the Cartesian images (as described above) helps remove the salt and pepper noise in the orientation gradient maps to produce robust fractures, they also remove any point singularities as well. However, visual inspection of the OSMs shows that the orientation preferences rotate continuously through ±180° along circular paths. This feature is characteristic of a pinwheel (Ohki et al., 2006). A better model of how the signal is smoothed in animal experiments could possibly help mitigate this problem. At this point, since our methodology does not obtain precise point singularities in the orientation gradient maps, we call these formations *pinwheel-like patterns*.

### Stability assessment

In order to measure the stability of functional map or RCM, a similarity measure between the synaptic conductance maps that reflect that particular structure in one developmental phase is compared against the synaptic conductance maps in another developmental phase based on Kullback–Leibler (KL) distance (Kullback, 1987). For example, the stability of the RCMs is determined by the least change in the KL distance. Mathematically, let *h*_1_(*w*) and *h*_2_(*w*) represent the two synaptic conductance histograms for synaptic conductance maps from two different developmental phases, *W*_1_ and *W*_2_. By normalizing the histograms, the synaptic conductance distribution functions are obtained as:

(16) $$p_{i}(w)=\frac{h_{i}(w)}{\max[h_{i}(w)]},\;i=1,2.$$

The similarity between synaptic conductance maps is then computed as:

(17) $$S(W_{1},W_{2})=\frac{1}{2}[\sum_{w}p_{1}(w)\log\left(\frac{p_{1}(w)}{p_{2}(w)}\right)+\sum_{w}p_{2}(w)\log\left(\frac{p_{2}(w)}{p_{1}(w)}\right)]$$

This KL measure provides a way to track changes in synaptic conductance during development and thus provides a measure of stability in the evolving functional maps or RCMs.

## Results

### Phase 1: early experience-independent OSM and ODM formation

The first phase of development begins with an *E-I* network that models the local recurrent microcircuit in layer 4 (Figure 1). This network consists of a 2-D lattice of 128 × 128 neurons with a 19 × 19 local neighborhood of synaptic connections to other *E* neurons (see Figures 2A,B). Neurons on the borders were assumed to have periodic boundary conditions such that topmost and bottommost neurons are regarded as neighbors, as are the leftmost and rightmost columns within each layer. The neurons exhibit spontaneous spiking activity (Chiu and Weliky, 2001; Huberman et al., 2006, 2008). This condition is simulated by injecting a constant small background current into a sub-population of both *E* and *I* neurons in layer 4 and LGN neurons of the network (see “Materials and Methods”). The conductance based synapses along with the recurrent connections within layer 4 ensures that the network is able to maintain this spontaneous activity (Kumar et al., 2008). The key aspect of the early experience-independent phase in our model is the absence of any retinal inputs (Figure 2B).

As the neurons in the network begin to spike due to spontaneous activity, STDP alters the synaptic strengths *w* and *z* (Equations 6, 7) and thus the connectivity between various neurons. Using our initial network model architecture (see “Materials and Methods”) combined with STDP in both excitatory and inhibitory synapses helps to achieve a good balance of excitation and inhibition. It is becoming more apparent that the ongoing balance of cortical excitation and inhibition plays a role in early development (Xing et al., 2011). The interesting aspect of our model is that inhibitory plasticity helps maintain this balance in the cortex that is qualitatively similar to observations in some recent experimental studies (Akerman et al., 2002)

**Figure 4:**
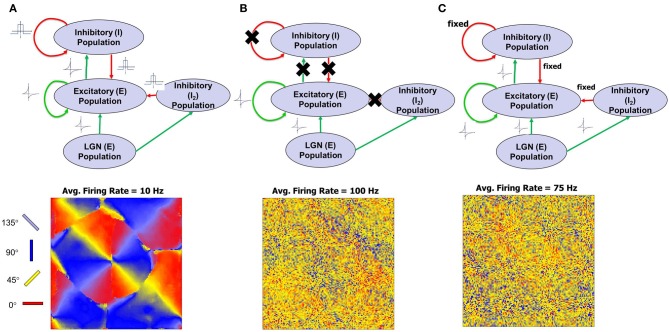
**Model architecture and simulations of first phase of recurrent cortical map (RCM) formation for three different cases are illustrated here.** The patterns of the RCM are aligned systematically with the Caretsian grid on which the population is laid out but the emergence of smooth organization is due to synaptic plasticity that changes the connectivity of neighbors to become similar to each other. **(A)** When STDP in both excitatory and inhibitory synapses are active, RCMs are formed with a clearly developing structure. The RCM is created using the *E* → *E* synaptic conductance values (“Materials and Methods”). The average firing rate of the *E* neurons of layer 4 is ~10 Hz. Low firing rates indicate a good balance between excitation and inhibition. **(B)** When there is no inhibition, RCMs fail to emerge and the average firing rate of the network is ~100 Hz. **(C)** In the case of fixed inhibition in the network, RCMs do emerge for some but not for all fixed inhibition settings. The average firing rate of the network for one such example setting of fixed inhibition for which no RCM emerges is ~75 Hz.

This aspect of development was tested by performing three types of experiments. In the first experiment, both *w* and *z* synapses obeyed STDP and in this case the network is able to operate at much lower firing rates (average of ~10 Hz). As *w* strengthens and create an imbalance in synaptic currents, *z* get rapidly potentiated due to an order-independent I-STDP where inhibition increases irrespective of the order of occurrence of pre- and post-synaptic spikes (Caporale and Dan, 2008) for small timing differences between pre- and post-synaptic spikes (Equation 7). This results in a rapid compensatory increase in inhibitory currents into the neurons in a self-organized manner thus effectively preventing the neurons from exceeding *V*_*T*_ more often (Vogels et al., 2011). This enables a good balance between excitation and inhibition and results in the emergence of RCM that have a locally smooth structure (Figure 4A).

In the second experiment all inhibitory synapses are turned off (i. e., *z* = 0). It was observed that firing rate of the *E* neurons was high on average (~100 Hz) but there was no emergent structure (Figure 4B). A high firing rate of neurons due to lack of inhibition forces the synapses to compete at a faster rate due to the asymmetric E-STDP rule (Equation 6) that results in a rapid rise in synaptic conductances for some synapses and a rapid fall in synaptic conductances for most others (due to a bias toward depression—see “Materials and Methods”). These rapid changes in synaptic conductance appear to be detrimental to the emergence of structure.

In third experiment excitatory synapses obeyed the STDP rule while plasticity was turned off for all the inhibitory synapses. Instead the inhibitory synapses were fixed (i. e., *z* = const) in synaptic strength. The exact setting for the fixed inhibitory synaptic conductance values was critical in order to see any emergence of structure. The firing rate was lower on average (~75 Hz) and was not conducive for the emergence of structure in most cases (Figure 4C).

**Figure 5:**
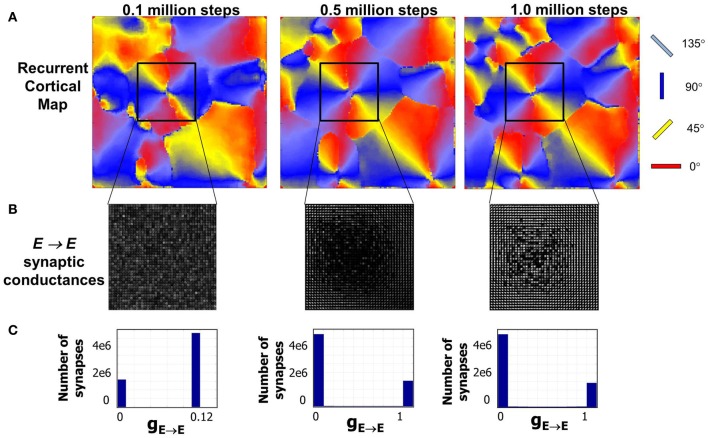
**Evolution of RCMs during Phase 1 at three different stages of development. (A)** After 0.1 million steps of simulation the RCM is well formed but not completely stable. The second stage after 0.5 million steps of simulation shows the emergence of structure in local synaptic connectivity between the *E* neurons in layer 4 (see “Materials and Methods”). The RCMs are stable after 1 million steps. **(B)** The synaptic conductances within the pin wheel for a 40 × 40 neighborhood (black square) is shown where there is a clear lack of appearance of structure after the first stage. After a 1 million steps of simulation, the RCM is well formed and the synaptic conductances show well developed structure except at the pin wheels. **(C)** The histogram of the synaptic conductances in the *E* → *E* synapses of layer 4 shows that the initial histogram of synaptic conductance values is bimodal with most of the synapses fixed at a value of 0.12. As the RCM evolves, this bimodal distribution changes due to competition between *E* → *E* synapses that is caused by STDP. This competition creates a sparse network with a majority of the synapses becoming zero while a fewer set of *E* → *E* synapses are fully potentiated to 1.0.

The emergence of RCM during the first phase of development is captured in Figure 5. The time taken for the emergence is just an artifact of the model parameter settings and reflects upon the fact that activity-dependent plasticity caused by STDP enables the emergence of RCMs (see “Materials and Methods”). The distribution of *E* → *E* synapse conductances (Figure 5A) shows smoothly varying structure in synaptic conductances within a neighborhood (Figure 5B). The distribution of synaptic conductances (Figure 5C) shows that the initial bimodal distribution becomes more separated and sparse in strong connections that is due to the competitive nature of STDP (Song and Abbott, 2001) and this competition between synapses that are pre-synaptic to neurons in layer 4 results in the modification of their synaptic connectivity and results in the emergence of RCMs.

**Figure 6:**
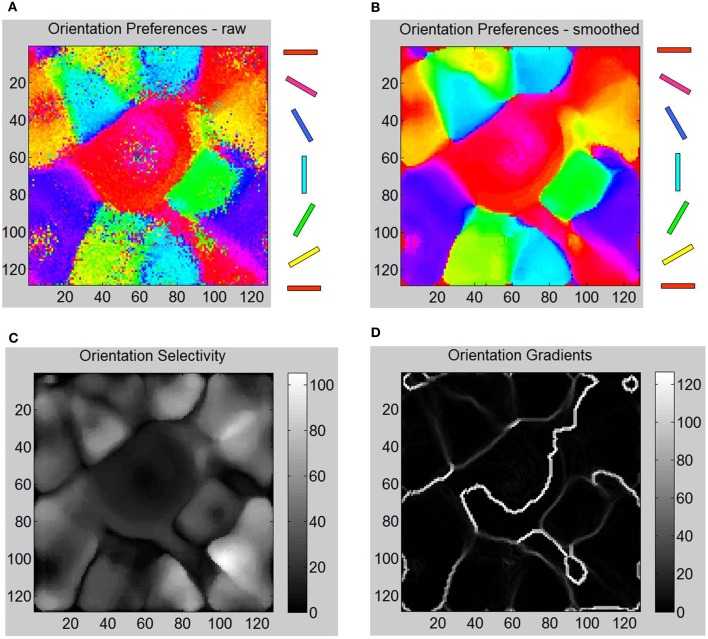
**Various aspects of formed OSM at the end of Phase 1 including orientation gradients, orientation selectivity, and orientation preferences of neurons are shown here. (A)** The oriented bar stimuli are provided as input to the LGN neurons and the raw firing rates of the *E* neurons in layer 4 are measured (“Materials and Methods”). **(B)** The smoothed responses (see “Materials and Methods”) of orientation preferences is computed and plotted in color. The oriented bars on the right provide the cyclic color code ranging from 0° to 180°. **(C)** Orientation selectivity of each *E* neuron is indicated using a grayscale map. The brighter colors indicate high selectivity where those *E* neurons in layer 4 respond sharply to a very narrow range of orientations and vice versa. The color scale shows the magnitudes of responses in a relative fashion. For example, neurons in the neighborhood of neuron at (110, 100) show strong selectivity (score of 110) to only 120° but not to other orientations while a neuron at (80, 50) shows a weak selectivity (score of 8) to all orientations including its preferred orientation of 150°. **(D)** The absolute magnitude of the orientation gradients at each *E* neuron is shown here. Here lighter values indicate high gradients (closer to 90°) while black indicates there neighborhoods have similar orientation preferences and thus no gradient at all. Orientation selectivity and orientation gradients are linked such that regions of high selectivity typically have low gradients while regions of low selectivity have high gradients in a manner qualitatively consistent with the data from the Blasdel paper. The discontinuous changes either occur alone (singularities), or they group together along lines (fractures). While there are many lines of fracture in this phase, there are no singularities, linear zones or pinwheels.

In order to test the orientation selectivity of *E* neurons in layer 4, the following experiment was performed (see “Materials and Methods”). The LGN neurons from both eyes were stimulated with oriented bar stimuli and the spiking activity of *E* neurons in layer 4 were measured in time windows of 10 ms and then averaged to compute their firing rates. The goal was to observe if there was preferential firing of V1 neurons to certain orientation of the bar stimuli presented in the LGN. A strong selectivity response feature required the adaptation of both the geniculocortical (LGN → *E*) and cortico-cortical (*E* → *E*) synapses such that the firing response of *E* neurons in layer 4 is strong to particular orientations and not others. The results show that the *E* neurons in layer 4 develop preferential firing to certain orientations and not to others (Figure 6). This illustrates that there was an emergence of OSMs with characteristic iso-orientation domains and fractures in the orientation gradient maps. However, there were no point singularities indicating the absence of any pinwheel-like formations. There were also no linear zones formed.

**Figure 7:**
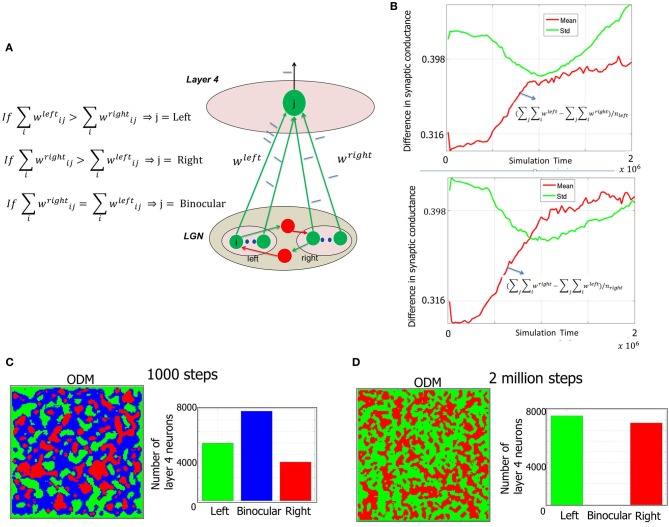
**Formation of ODMs during Phase 1. (A)** The methodology of constructing the ODMs is outlined here (see “Materials and Methods”). The synaptic conductance changes induced due to STDP at the geniculocortical synapses from the LGN neurons corresponding to each eye are tracked over time. If the *E* neuron in layer 4 has a stronger set of afferents from the LGN from the left eye compared to the right eye, then the *E* neuron is labeled as “left” and color coded as green in the ODM. The exact opposite scenario results in the *E* neuron labeled as “right” and color coded as red in the ODM. If there is a tie (as in the beginning), then the *E* neuron is labeled as “binocular” and color coded as blue in the ODM. **(B)** The sum of the synaptic conductances from the LGN neurons corresponding to the left eye is compared against the sum of the synaptic conductances from the LGN neurons corresponding to the right eye at each *E* neuron. Each of these neurons are labeled as “left” or “eye” as described above. Then the mean and standard deviation of the difference between left and right eye afferent synapses for the neurons (*nleft*) labeled “left” is computed and plotted in a semilog format with the ordinate plotted in log scale while the abscissa data is plotted in regular scale. It can be seen that the mean value of the difference increases slowly. However, the standard deviation of the difference is higher than the mean implying that many of the *E* neurons have a very small difference in synaptic conductances while a few have a much larger difference. Similar behavior was observed for the right eye as well. This measure shows that the ODMs are not really stable during the Phase 1 in our model. **(C)** Early ODM appears to have several binocular *E* neurons since all the geniculocortical synapses are initialized with the same synaptic strength and there have not been sufficient inputs to alter the synaptic strengths via STDP. **(D)** At the end of Phase 1, the *E* neurons in the ODM shows eye selectivity with an even split of neurons becoming selective to one of the two eyes. There are no more binocular neurons. The ODM, however, appears fragmented with no large contiguous areas of neurons showing preference to one eye and not the other. Instead it has lots of small contiguous areas of various sizes. This is due to random stimulation of the LGN neurons with background activity with no temporal or spatial contiguity during Phase 1. The STD and mean data in **(B)** also provides further support to this basic phenomenon during this phase. However, this improves during the second and third phases of development as shown in Figures 10, 12 when there is more structure in the input data.

The feedforward inputs triggered by spontaneous background spiking activity in the LGN results in the excitation of layer 4 neurons via the geniculocortical synapses (i.e., LGN → *E* synapses). The synaptic inputs originating from LGN can be partitioned into two groups based on the origin of LGN neurons: left and right eyes (Figure 7A). Initially the synapses are all of equal strength (i.e., no bias) such that the *E* neurons in layer 4 respond equally to spikes from LGN neurons corresponding to both the eyes (Figure 7C). However, noisy background spiking activity in LGN causes STDP to select some synapses to potentiate while others to depress depending on temporal correlations among the spikes that impinge on the *E* neurons in layer 4. This process results in introducing a bias in the synaptic strengths from LGN → *E* neurons in layer 4 due to STDP induced competition among all geniculocortical synapses at any given *E* neuron. As a result, the strength of geniculocortical synapses from one eye ends up being more than from the other eye (Figure 7A) and thus *E* neurons in layer 4 begins to develop eye selectivity. The eye selectivity of *E* neurons across layer 4 manifests as an ODM (Figure 7D). This early experience-independent formation of ODM while balanced (i. e., number of *E* neurons that are selective to the left and right are equal) is still fragmented without any contiguous patches of neurons as found in the visual cortex of several species. This is because in our model, the LGN neurons are primarily stimulated via background activity that has no temporal or spatial contiguity during Phase 1. The mean value of the difference in synaptic conductance between the geniculocortical synapses from both eyes increases slowly (Figure 7B). However, the standard deviation of the difference is higher than the mean implying that many of the *E* neurons have a very small difference in synaptic conductances while a few have a much larger difference. This measure shows that the ODMs are not really stable during the Phase 1.

### Phase 2: late experience-independent ODM and OSM refinement

**Figure 8:**
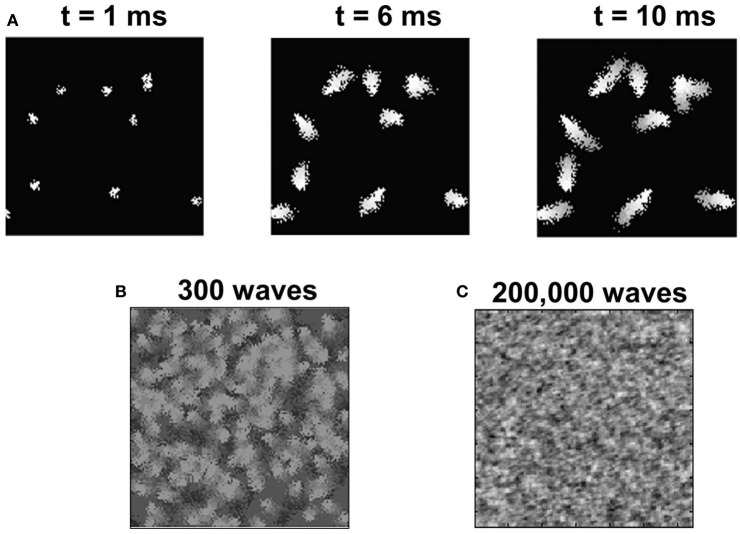
**The retinal wave model. (A)** Shows 128 × 128 RGCs from one retina. After 1 ms of spontaneous firing of RGCs, there are 10 sites that are randomly initiated to generate spikes. These sites create further activity among neighboring RGCs to propagate a wave of spikes as shown for *t* = 6 ms and *t* = 10 ms. Each such spontaneously initiated wave activity is terminated at the end of 10 ms. The 128 × 128 retinal wave image is donw-sampled to a 48 × 48 image and provided as input to the LGN. **(B)** The superposition of all spike activity after 300 retinal waves shows a good spatial distribution of spike activity covering all parts of the retina. **(C)** The late experience-independent phase in our model lasts for around 200,000 retinal waves when all the RGCs in the retina are activated at least once and the spiking activity of RGCs resembles a Gaussian random field-like distribution.

In the second phase of development, in addition to the basic thalamocortical circuit of Phase 1, the LGN is now connected to two groups of RGC corresponding to the two eyes (Figure 1). The RGC inputs to LGN represent internally generated spikes in the form of retinal waves (“Materials and Methods”) that provide a robust signal to drive activity in both the LGN and in layer 4 of V1. Such spontaneous activity has been implicated in the development of ODMs and retinotopy in V1 (Mooney et al., 1996; Butts, 2002; Godfrey and Swindale, 2007; White and Fitzpatrick, 2007; Huberman et al., 2008; Feller, 2009). An example of a retinal wave generated for a given eye with *N* = 10 is shown in Figure 8A. The retinal waves after 300 and 200,000 waves are shown in Figures 8B,C, respectively. It can be noted that by the end of 200,000 waves, all the RGCs have been selected as initiation sites and the distribution of RGC activity resembles a Gaussian random field.

**Figure 9:**
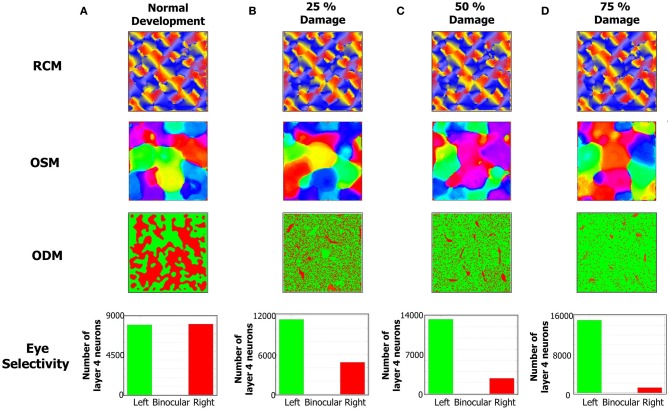
**The formation of RCMs, OSMs, and ODMs under normal development and with lesion in the LGN → *E* pathway is shown here.** The RCMs are the most stable since they are fully dependent on local recurrent connections within layer 4. The OSMs are altered more since their formation is dependent on both local recurrent connections as well as feedforward connections from LGN to layer 4. The ODMs are the most affected by lesions since they are primarily affected by LGN to layer 4 connections. **(A)** The column shows the RCM, OSM, and ODM after 4 million steps. The eye selectivity of the *E* neurons in layer 4 appears to be evenly balanced between the two eyes. The ODM appears to have more contiguous patches of neurons that respond to only one of the two eyes. The OSM is developed with more patches of iso-orientation domains. **(B)** The development of ODMs is affected with more *E* neurons tuned to the left eye compared to the right eye when 25% of RGCs in the right eye are prevented from stimulating the right LGN neurons. The OSMs also show a reorganization of the map albeit without any dramatic changes. The RCMs are still stable. **(C)** The development of ODMs is more severely affected compared to **(B)** when 50% of RGCs in the right eye are prevented from stimulating the right LGN neurons. The OSMs still show iso-orientation domains but there is a re-organization of the patches relative to the 25% lesion case. **(D)** The development of ODMs is even more severely affected compared to **(C)** when 75% of RGCs in the right eye are prevented from stimulating the right LGN neurons. The RCMs on the other hand do not appear to be that dramatically affected. The OSMs still show some differentiation in its orientation preferences albeit undergoing more re-organization compared to the 50% lesion case.

In this model, the spikes generated by retinal waves are transmitted via geniculocortical synapses to activate the *E* neurons in layer 4. It should be noted that our model assumes that in this phase of development, there is no separation of RGCs into ON and OFF ganglion cells (Myhr et al., 2001; Huberman et al., 2008). The distribution of the geniculocortical synapses to *E* neurons is sharpened further due to STDP and the resulting ODM shows more distinct patches selective to a given eye compared to ODMs in Phase 1 (Figure 9A).

The emergence of ODMs was also analyzed based on lesion studies. There are animal studies that suggest, for example, that retinal wave disruption caused by intraocular injection of epibatidine reduces firing of the RGC thereby affecting the development of eye-specific retinogeniculate projections and eventually the development of functional maps as well (Wong, 1999; Feller, 2009). In our model, we qualitatively simulate the disruption of retinal waves by cutting off neural activity in a percentage of neurons in the right eye of the LGN. The resulting RCMs, OSMs, and ODMs (Figures 9B–D) are compared against the case of normal development (Figure 9A). In our model, this bias emerges due to the competitive nature of the STDP rule. When more inputs are received from the left eye, there is a higher probability for the geniculocortical synapses from the left eye to cause a post-synaptic spike. This in turn implies that the geniculocortical synapses from the left eye are going to potentiate a lot more than those from the right eye and thus the *E* neurons in layer 4 become more selective to spikes from the left eye.

RCM development is only affected in a minor fashion due to lesions to the LGN cells because RCM formation is primarily dictated by the *E* → *E* synaptic conductance maps while ODM and OSM formation is dictated by both LGN → *E* and *E* → *E* synapses. Since the changes in inputs from retinal waves drive the geniculocortical synapses, the spikes in the *E* neurons in layer 4 are primarily caused by the LGN. So, the synaptic changes in geniculocortical synapses (which affects ODM and OSM) are more dramatic than in the cortico-cortical synapses (which affects OSM and RCM).

**Figure 10:**
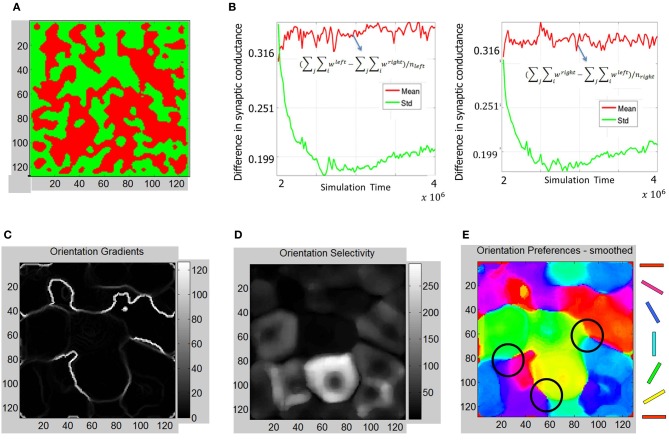
**ODM and OSM formation during Phase 2. (A)** The ODM appears to have large more contiguous patches of eye selectivity compared to the output after Phase 1. **(B)** This maturity in ODM formation is caused by more stability in the divergence between the mean and standard deviation calculations in the semilog plots shown here. This divergence is very clear unlike in Figure 7. The mean and standard deviation also seem to stabilize in the later parts of Phase 2. **(C)** The absolute magnitude of the orientation gradients at each *E* neuron shows singularities and fractures. Here white indicates high gradient values while black indicates no gradient at all. The gradients image show several fractures in the data. The average fraction of the total synaptic drive at each *E* neuron selective to a given eye was also calculated. For example, for the “left” *E* neurons, (∑_*i*_ *w*^left^ − ∑_*i*_ *w*^right^)/(∑_*i*_ *w*^left^ + ∑_*i*_ *w*^right^), was ~71%. Similarly, the fraction was ~70% for the “right” *E* neurons at the end of Phase 2. **(D)** Orientation selectivity shows brighter colors that indicate high selectivity with those *E* neurons in layer 4 respond to a very narrow range of orientations and vice versa. The color scale shows the magnitudes of responses in a relative fashion. For example, neurons in the neighborhood of neuron at (85, 80) show strong selectivity (score of 270) to only 30° but not to other orientations while a neuron at (60, 60) shows a weak selectivity (score of 3) to all orientations including its preferred orientation of 60°. **(E)** The smoothed orientation preference map shows iso-orientation domains and three weakly formed pinwheels marked by the three black circles. We call these weakly formed pin wheels since they are not corroborated by singularities in the orientation gradient maps. This is because the smoothing operation on the Cartesian images removes the spurious edges created by noisy neuron responses while also removing any trace of the singularities as well. However, close inspection shows that there are three locations marked with black circles where the orientation preferences rotate continuously through ±180° along circular paths. We refer to these patterns as a pinwheel-like pattern. It should be noted that there are no clear appearance of point discontinuities in the orientation gradient maps to corroborate the pin-wheel centers within these pinwheel-like-patterns that clearly appear in animal data.

The ODMs formed in Phase 2 show more contiguous patches of eye selectivity (Figure 10A) compared with Phase 1. The mean and standard deviation of difference in synaptic conductances (Figure 10B) show a more clear separation on the semi-log plot indicating that the ODM formed in Phase 2 is more stable than in Phase 1. The orientation tuning of *E* neurons in layer 4 during Phase 2 was evaluated by stimulating the LGN neurons for both eyes using the same oriented bar stimuli (Figure 6) during Phase 1. The results show that the *E* neurons in layer 4 develop more well-defined iso-orientation domains (Figure 10E) and fractures (Figure 10C) in Phase 2 compared to Phase 1. This change is caused by STDP due to a spike inputs from the geniculocortical synapses in Phase 2 due to the retinal waves that have more spatial and temporal contiguity compared to noisy background level activity in Phase 1. The peak firing response of *E* neurons in layer 4 are on the average larger than in Phase 1 as indicated by the larger dynamic range in orientation selectivity maps (Figure 10D). This shows that the *E* neurons in Phase 2 have developed sharper orientation selectivity and the OSM is actively evolving due to activity-dependent plasticity. There are also three clear pinwheel-like patterns that emerge in this stage as depicted in black circles (Figure 10E) where the orientation preferences rotate continuously through ±180° along circular paths. However, there were no corresponding point singularities that could be extracted from the orientation gradient due to the limitations of the methodology used (see “Materials and Methods”).

### Phase 3: experience-dependent OSM and ODM refinement and maintenance

**Figure 11:**
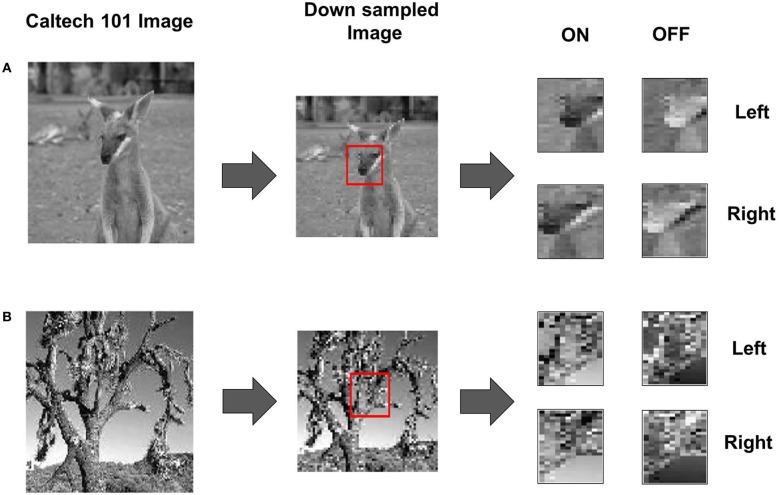
**Natural stimuli during Phase 3. (A)** The input image from the Caltech database is down sampled from a 320 × 200 image to a 128 × 128 image and pixels from within a 48 × 48 window from the center of the sub-sampled image (red box) are used to create the stereo pair. The resulting image for the right eye is obtained by shifting the fovea to the right and applying a scaling factor of 1.025 on the left image. The resulting stereo pair is finally processed to generate ON and OFF images for each eye (see “Materials and Methods”). **(B)** A second example of an image from the Caltech 101 database using the same scaling and pixel shift as in **(A)** is used for extracting the down sampled ON and OFF images. The appearance of the pixels in the ON and OFF images for both examples has similar statistics in terms of the contrast and oriented edges.

For the third phase of the developmental process, the model is exposed to sensory experience in the form of images from a database of real-world images from the Caltech 101 database (see “Materials and Methods”) to study the effects of experience-dependent plasticity (Yao and Dan, 2001) on the refinement of OSMs and ODMs. In our model, the RGCs in this phase are assumed to be segregated into ON and OFF neurons as evident in animals after eye opening (Koehler et al., 2011). The ON and OFF regions each contain 48 × 24 neurons. The ON neuron responses are encoded as spikes (see “Materials and Methods”). The OFF neuron responses are computed by taking the inverse of the ON image and then rectifying the image so that there are no negative values (Figure 11). The resulting image is encoded as spikes (Equation 11).

**Figure 12:**
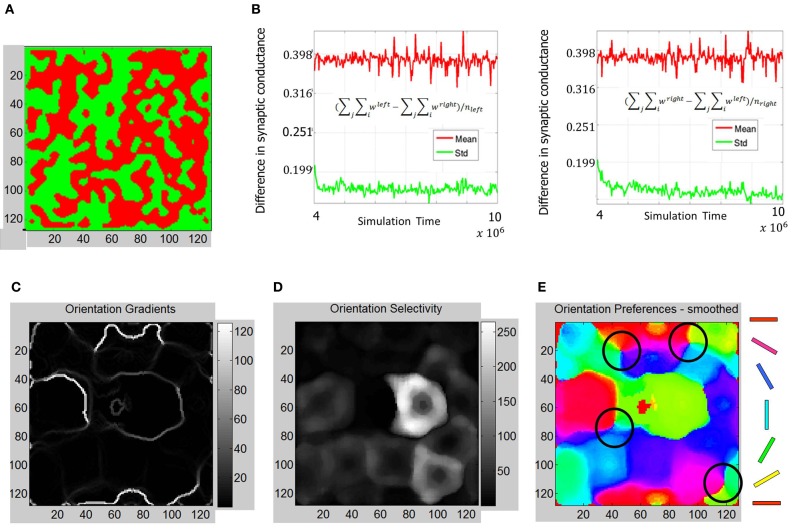
**Functional maps summary and orientation selectivity of neurons in OSMs during Phase 3. (A)** Comparison of functional map development during all three phases shows the progressive refinement of the ODM, RCM, and OSM. The ODM is stable and shows well-defined and contiguous patches of eye selectivity. **(B)** The mean and standard deviation parameters as described in Figures 7, 10 show more divergence and stability compared to Phase 2 The average fraction of the total synaptic drive for the “left” *E* neurons was ~123% and ~124% for the “right” *E* neurons at the end of Phase 3. **(C)** The resulting orientation gradient maps show well-defined fractures but no singularities. **(D)** The orientation selectivity has similar characteristic to that of Phase 2 except that the peak magnitude of selectivity showing more sharpness (i.e., higher magnitude). **(E)** The orientation preference maps show clearly defined iso-orientations and four weakly formed pinwheels marked by four black circles.

The ODMs formed in Phase 3 show contiguous patches of eye selectivity (Figure 12A) in a manner similar compared with Phase 2. The mean and standard deviation of difference in synaptic conductances (Figure 12B) show an even more clear separation on the semi-log plot indicating that the ODM formed in Phase 3 is more stable than in Phase 2. The results also show that *E* neurons in layer 4 are more finely tuned to particular orientations compared to Phase 2 (Figure 12E). The dynamic range of the orientation selectivity map (Figure 12D) is larger than in Phase 2. There are four distinct pinwheel-like patterns that emerge after learning in Phase 3 (Figure 12B) but do not have any point discontinuities in the orientation gradient maps at the centers of these pin wheel patterns. The ODM maps are also more refined and develop noticeable contiguous regions of eye selectivity. The interesting aspect here is that the RCMs seem to have stabilized in Phase 2 and appear qualitatively similar between Phase 2 and Phase 3 while the ODM and OSM continue to undergo refinements after exposure to the images from the Caltech 101 database for over 6 million steps. This is qualitatively consistent with some experimental observations (Chapman et al., 1996; Crair et al., 1998) where the orientation tuning responses change with environmental stimuli.

### Stability of functional maps

**Figure 13:**
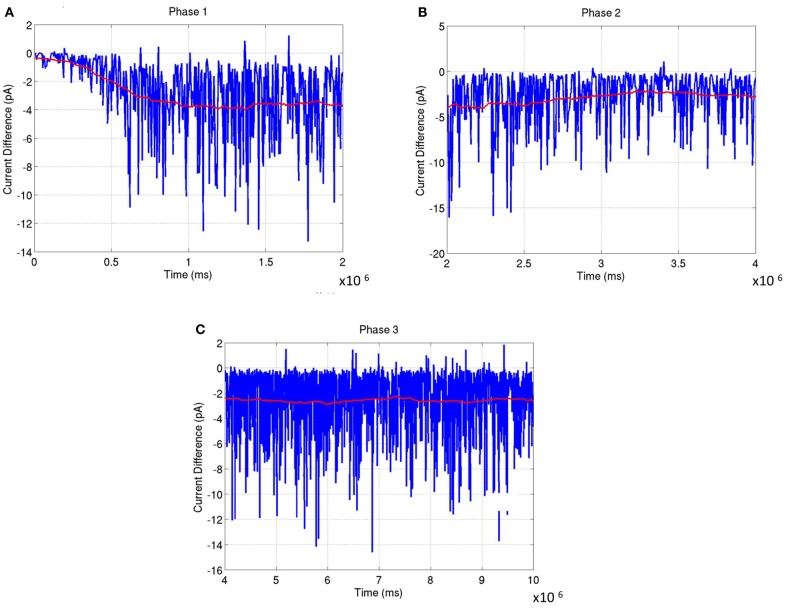
**The average synaptic current difference between excitation and inhibition is plotted as a function of developmental time. (A)** Experience-independent OSM development (Phase 1). **(B)** Experience-independent ODM development (Phase 2). **(C)** Experience-dependent refinement of both OSM and ODM (Phase 3). In all the plots, the instantaneous current difference at each time step is shown in blue while the average current difference is shown in red. The plots show that the stability of the functional maps are correlated closely to the fact that the average current differences become progressively smaller as the maps develop. This is enabled by inhibitory plasticity and helps in preventing any rapid changes in synaptic conductances.

The network was analyzed for stability across all three phases of development. In Phase 1, the balance in synaptic currents driving neurons of layer 4 for this phase of development was measured by computing the difference between excitatory and inhibitory currents at each *E* neuron by averaging across a time window of 200 ms. Simulations show that the net current during this phase moves from an initial bias toward excitation to a more negative bias toward inhibition that slowly reaches a steady state value (see red line in Figure 13A). The negative bias shows that on average the influence of inhibition is stronger than excitation so as to compensate for the imbalance in the number of *E* to *I* neurons in layer 4. This dynamic ensures that the firing rates of the neurons are low and conducive for learning the RCMs and functional maps.

**Figure 14:**
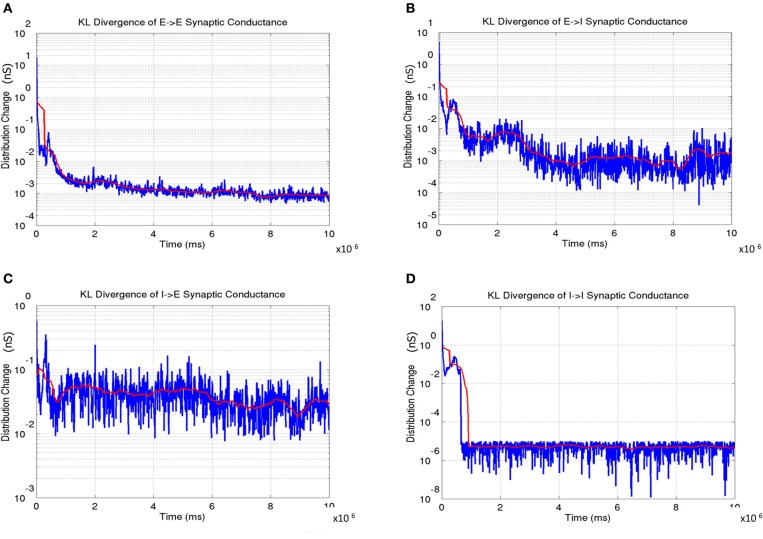
**The change in synaptic conductance distributions during the course of learning. (A)** The *E* → *E* change shows a gradual decrease in the change with the lowest values in the third phase indicating stability in the formed OSM and ODMs. **(B)** The *E* → *I* change shows a similar trend as in **(A)**. **(C)** The *I* → *E* change also shows gradual stabilization as a function of development. **(D)** The *I* → *I* change achieve stability very early and remain very stable after indicating that the synaptic conductances between inhibitory interneurons are stabilized rapidly compared to the other three types of synapses. In all the plots, the instantaneous change in synaptic conductance distributions at each time step is shown in blue while the average change is shown in red.

The distribution of synaptic conductances was also tracked dynamically throughout the learning process by computing the difference between the normalized distribution of synaptic conductances (*E* → *E*, *E* → *I*, *I* → *E*, and *I* → *I* as shown in Figure 14) using the KL measure (Equation 17) once every 5000 simulation steps (or 5 s). This dynamic captures a measure of stability in the learning process during development. The results show that all the four types of conductances slowly reach a steady state at the end of Phase 1 with the KL divergence showing very small fluctuations.

The network exhibits a better balance between excitation and inhibition progressively as a function of development (Shapley et al., 2003; Okun and Lampl, 2008) (see Figure 13). The stability of the formed RCMs in the second phase of development is measured using KL divergence in synaptic conductances (Figure 14). The results show that the network is progressively more stable during development since the KL divergence is lower progressively for all four types of synaptic conductances in layer 4.

The OSMs and ODMs undergo refinement throughout the three phases of development. The primary cause for the refinement is the change in the nature of LGN inputs from random activity in Phase 1 to retinal waves in Phase 2 to natural stimuli in Phase 3. At the end of Phase 3 (i. e., after over 5.5 million steps of natural image stimuli) the OSMs and ODMs appear to stabilize such that the orientation responses of neurons in layer 4 no longer shift as observed earlier (compare Figures 6, 10, and 12).

**Figure 15:**
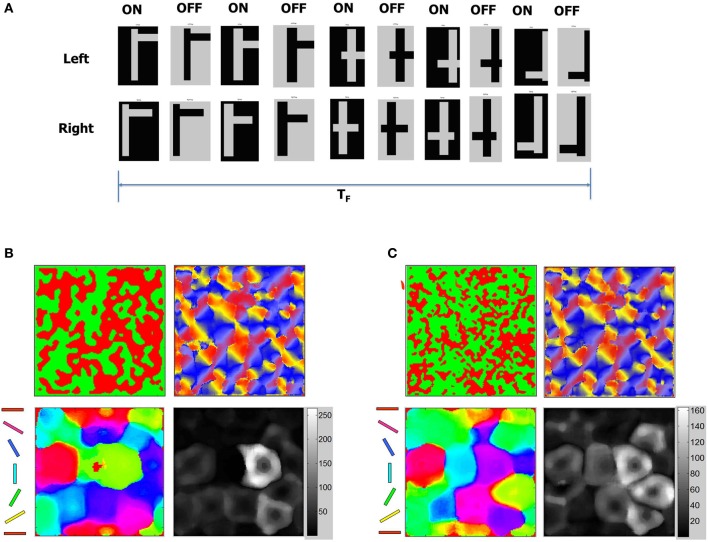
**ODM and OSM change in response to new stimuli. (A)** Stability of the development process was tested using a sequence of test patterns shown here as stereo pairs with ON and OFF images for each pair. The test patterns consist of mixtures of horizontal and vertical lines that combine to form flag-like patterns. These flag-like patterns are presented for total duration of *T*_*F*_ seconds. The duration of each flag-like pattern was between 10 and 100 ms. **(B)** OSM, RCM, and ODM maps after presenting the flag patterns for a duration of *T*_*F*_ = 10 s after Phase 3 shows that the maps are stable (compared to Figure 12) despite constant variations in the duration of presentation of each flag-like patterns. **(C)** OSM, RCM, and ODM maps after presenting the flag patterns continuously for a duration of *T*_*F*_ = 5000 s after Phase 3 shows that the ODM and OSM change while RCM does not change much at all (compared to panel **B**). The change in orientation selectivity in the early and late stage of input patterns shows a noticeable change in the OSMs. The number of neurons with responses close to 0° or 180° (red and magenta) and 90° (cyan and green) have gone up compared to the plot in panel **(B)** indicating that the change reflects the dominance of vertical and horizontal bars in the stimuli.

It is known that OSMs and ODMs continue to undergo noticeable refinements if there is substantial change in the input environment (Blakemore and Cooper, 1970; Sengpiel et al., 1999; Krelle et al., 2011). We conducted an experiment to verify if this occurs in our model. We created a set of new flag patterns primarily consisting of horizontal and vertical bars (Figure 15A). This stimulus is considerably different from the Caltech 101 database images since they do not provide contrast information in any other direction except 0° or 90°. These inputs stimuli were provided as stereo inputs to the LGN (“Materials and Methods”). Despite repeated presentations of such stimuli ranging from short (*T*_*F*_ < 1 s) to longer durations (*T*_*F*_ = 10 s), the OSMs and ODMs are not affected and remain stable (Figure 15B). This is due to an exquisite balance in currents created by the E-STDP and I-STDP plasticity mechanisms where any instantaneous imbalance in currents is rapidly compensated by plasticity to restore the balance (Figure 13).

If there is a volley of high-frequency spikes due to stimuli during such short durations that cause the *E* neurons to spike more rapidly, the inhibitory plasticity for both feedforward and recurrent synaptic connections enables the system to increase the synaptic conductance *z* of the inhibitory synapses. This results in increased inhibitory currents to the active *E* neurons thereby removing the imbalance (Vogels et al., 2011). Thus E-STDP and I-STDP enables the stable maintenance of OSMs and ODMs despite continuous plasticity.

We also applied the same flag patterns with horizontal and vertical bars for a very long duration (*T*_*F*_ = 5000 s). The OSMs and ODMs do see changes (Figure 15C) after this long exposure reflecting that only prolonged and consistent presentations of stimuli can change the orientation tuning response of the *E* neurons in layer 4. This change is again possible due to activity-dependent STDP that slowly changes the tuning properties of *E* neurons during the small windows of opportunity where *E* neurons in layer 4 spike despite operating in a well-balanced regime between excitation and inhibition. Since these spikes are sparse, our model requires prolonged periods of stimuli exposure to effect changes in the stabilized ODM and OSM. These results qualitatively agree with experimental results (Blakemore and Cooper, 1970; Sengpiel et al., 1999; Krelle et al., 2011) that suggest that influence due to environmental stimuli can affect functional maps in V1. It also shows that the number of *E* neurons that respond to 90° (blue regions) or 0° (red regions) increases by 25% or more compared to OSM at the end of 10 million steps (Figure 15C). This is also qualitatively consistent with observations in the visual cortex (Sengpiel et al., 1999). It is interesting that RCMs are not affected much because geniculocortical synapses are affected more by new inputs from LGN compared to cortico-cortical synapses.

## Discussion

The proposed spiking model is the first to cover the three developmental phases during the formation of OSMs and ODMs with continuous synaptic plasticity in the form of STDP. The model offers a biologically plausible explanation for this formation in which E-STDP and I-STDP at the excitatory and inhibitory synapses, respectively combine to enable the development and maintenance of these functional maps. This is consistent with recent models that suggest that cortical reorganization is reliant on spike timing (Song et al., 2000; Young et al., 2007). It is also consistent with a recent model that suggests that inhibitory plasticity could play a key role in the formation and maintenance of functional cortical circuitry (Vogels et al., 2011).

It is possible for our model to demonstrate OSM and ODM formation directly via external simulations (i.e., experience) and skipping the two experience-independent phases. However, our simulations demonstrate that STDP based learning driven by spontaneous intra-cortical spiking activity can result in the formation of orientation maps and ocular dominance maps as suggested by prior research (Huberman et al., 2006; White and Fitzpatrick, 2007). In this early experience-independent phase ODMs and OSMs appear due to spontaneous activity between LGN neurons and the *E* neurons in layer 4. In the late activity-independent phase where the spontaneous activity in the LGN is generated via retinal waves (Butts, 2002; Godfrey and Swindale, 2007), the ODMs appear to become selectively tuned to one of the two eyes due to competition induced by E-STDP between geniculocortical synapses (Feller, 2009) thus dividing the cortical layer 4 into ocular dominance patches. The important point here is that the model qualitatively shows that there is no need for external input to enable the development of ODMs or OSMs as long as there is STDP driven plasticity in both *E* and *I* synapses. The influence of external inputs, however, does improve the sharpness of the tuning responses in V1.

However, the model in this study should be considered a simple model that is biologically incomplete in its complexity. It does not consider the development of retinogeniculate synapses (Chen and Regehr, 2000; Feller, 2009) nor does it model the differential sensitivities of the ON and OFF cells in their response to variations in contrast relative to mean luminance (Zaghloul et al., 2003). It also does not consider other types of plasticity found in biology such as short term plasticity (Tsodyks and Markram, 1997; Tsodyks et al., 1998) that affects population dynamics during different functional states (Mark and Tsodyks, 2012) and homeostatic plasticity (Turrigiano and Nelson, 2004; Watt and Desai, 2010). It also does not model the complexity in structure and function of neurons including multiple compartments (Hodgkin and Huxley, 1952; Izhikevich, 2004) and other forms of plasticity such as dendritic plasticity and its relation to STDP (Williams et al., 2007; Sjostrom et al., 2008; Froemke et al., 2010). It should be noted that in this model, neurons are assumed to be mature from early stages of development. However, in reality there are immature depolarizing neurons in the early stages of development that are characterized by a high concentration of Cl^−^ ions (Hensch, 2005). This aspect of development is not considered in this model.

The model is currently being extended to account for the development of direction selectivity. It is well known that some neurons in the visual cortex are selective to direction of motion of visual stimuli (Weliky et al., 1996; White and Fitzpatrick, 2007). Furthermore, direction selectivity map (DSM) formation appears to lag behind OSM in its formation (Li et al., 2006). Recent physiological evidence suggests that the DSMs in the cortex are nested geometrically within OSMs such that an iso-orientation domain is subdivided into a pair of smaller domains that represent opposite directions of stimulus motion (Kisvarday et al., 2001; White and Fitzpatrick, 2007). Finally, there is also mounting evidence that activity-dependent plasticity such as STDP enables the formation of DSMs (Fiser et al., 2004; Carver et al., 2008; Markram et al., 2011) and recent models show the possibility of forming DSMs using STDP (Buchs and Senn, 2002; Wenisch et al., 2005). Thus a natural extension of the proposed model is to account for the formation of DSMs using STDP within the context of development of all other functional maps such as OSMs and ODMs.

In summary, the present study developed a simple model of a thalamocortical circuit with an initial unstructured map topology that is refined using continuous plasticity in a self-organized fashion to form RCMs, OSMs, and ODMs based on neural activity during three phases of development: endogenously generated cortical activity, followed by activity that arises endogenously in the form of retinal waves and finally activity evoked during sensory experience. Continuous plasticity based on STDP in both excitatory and inhibitory synapses serves as the key mechanism for the development, refinement, and stable maintenance of the formed maps and could also serve as a basis for the development of other functional maps such as DSMs.

### Conflict of interest statement

The authors declare that the research was conducted in the absence of any commercial or financial relationships that could be construed as a potential conflict of interest.
